# Thermoelectric and Transport Properties of Delafossite CuCrO_2_:Mg Thin Films Prepared by RF Magnetron Sputtering

**DOI:** 10.3390/nano7070157

**Published:** 2017-06-27

**Authors:** Inthuga Sinnarasa, Yohann Thimont, Lionel Presmanes, Antoine Barnabé, Philippe Tailhades

**Affiliations:** CIRIMAT, Université de Toulouse, CNRS, INPT, UPS, 118 route de Narbonne, F-31062 Toulouse CEDEX 9, France; sinnarasa@chimie.ups-tlse.fr (I.S.); presmanes@chimie.ups-tlse.fr (L.P.); barnabe@chimie.ups-tlse.fr (A.B.); tailhades@chimie.ups-tlse.fr (P.T.)

**Keywords:** thermoelectric, oxides, delafossite, thin film, power factor, degenerated semiconductor, hopping mode

## Abstract

P-type Mg doped CuCrO_2_ thin films have been deposited on fused silica substrates by Radio-Frequency (RF) magnetron sputtering. The as-deposited CuCrO_2_:Mg thin films have been annealed at different temperatures (from 450 to 650 °C) under primary vacuum to obtain the delafossite phase. The annealed samples exhibit 3R delafossite structure. Electrical conductivity σ and Seebeck coefficient S of all annealed films have been measured from 40 to 220 °C. The optimized properties have been obtained for CuCrO_2_:Mg thin film annealed at 550 °C. At a measurement temperature of 40 °C, this sample exhibited the highest electrical conductivity of 0.60 S·cm^−1^ with a Seebeck coefficient of +329 µV·K^−1^. The calculated power factor (*PF* = *σS*²) was 6 µW·m^−1^·K^−2^ at 40 °C and due to the constant Seebeck coefficient and the increasing electrical conductivity with measurement temperature, it reached 38 µW·m^−1^·K^−2^ at 220 °C. Moreover, according to measurement of the Seebeck coefficient and electrical conductivity in temperature, we confirmed that CuCrO_2_:Mg exhibits hopping conduction and degenerates semiconductor behavior. Carrier concentration, Fermi level, and hole effective mass have been discussed.

## 1. Introduction

The thermoelectricity is a promising technique to overcome the issues in recovering waste heat to electricity without using moving parts. Thermoelectric (TE) effect is defined as the conversion of a temperature gradient directly into electricity and vice versa [1]. Thermoelectric generators have several advantages: they are particularly reliable, maintenance free, and durable with long operating life under extreme conditions [2].

The performance of the TE materials is described by the dimension less figure of merit *ZT* [1].

(1) $$ZT=\frac{\sigma S^{2}}{\left(K_{e}+K_{\mathrm{th}}\right)}\times T$$

In these equations, *σ* and *S* are respectively the electrical conductivity and the Seebeck coefficient [3] at the given temperature *T* and *K*_e_ and *K*_th_ are respectively due to the electron transport and the lattice phonon at the given temperature *T*. The enhancement efforts of *ZT* is very challenging because of the *σ*, *S*, and *K*_e_ interdependence. Indeed, as the carrier density expands, the electrical conductivity increases and at the same time it reduces the Seebeck coefficient and increases the electronical thermal conductivity. To optimize TE materials, the Power Factor (*PF* = *σS*^2^) must be increased without increasing the global thermal conductivity. This can be done by improving the carrier concentration and the mobility and/or reducing the lattice thermal conductivity *K*_th_ by introducing scattering centers with point defects [4], interfaces [5], and nanostructuration [6].

The advantage of the thin films versus bulk materials is rarely guested with the *PF*. Only the Figure of Merit (*ZT*) can reveal it. In fact some macroscopic disorder can lead to a significant reduction of the thermal conductivity of thin films relative to that observed in bulk material [7,8,9]. As *ZT* takes into account the thermal conductivity, thin films could have better TE properties than the bulk thanks to their microstructures. Moreover, the low dimension system obtained in thin films could make a significant band structure modification that could impact the Seebeck coefficient.

Transition metal oxides (TMOs) are a captivating class of materials due to their wide ranging electronic, chemical, and mechanical properties. Furthermore, they are gaining increasing attention for their thermoelectric (TE) properties due to their high temperature stability, tunable electronic and phonon transport properties, and well-established synthesis techniques.

Delafossite type oxides Cu^I^M^III^O_2_ with M = (Fe, Al, Ga, Cr…) due to their large range of properties and the abundance of their constituent elements in the nature, have been studied for several applications such as transparent p-type conducting oxides (TCO) [10,11,12,13,14,15,16,17], transparent electronic devices [18,19,20,21,22,23,24,25], dye-sensitized solar cells [26,27,28,29], and photoelectrodes [30] but also for outstanding catalysis [31] and photo-catalysis [31,32,33,34,35,36,37,38,39,40,41], antibacterial [42], luminescence [43,44,45], gas and temperature sensing [46,47,48,49], magnetic and electric [50,51,52,53,54], energy storage [55], oxygen storage [56], water reduction [57], thermoelectricity and superconductivity [58] properties. In the oxide family, the cation Cu^I^ is a monovalent metal and the cation M^III^ is a trivalent metal. Delafossite structure can be described as a stack of cation Cu^I^ layer and MO_6_ octahedron layer along c axis. Each cation Cu^I^ is linearly coordinated to two oxygens belonging to upper and lower MO_6_ octahedron. Even if Cu-based delafossite type oxides receive most of the attention for their optoelectronic properties as a p-type transparent semiconductor, they exhibit also interesting thermoelectric (TE) properties [59,60,61,62,63,64,65,66,67].

Among the Cu-based delafossites, CuCrO_2_ is currently of interest due to its attractive physical properties for the applications mentioned above [68,69,70,71,72,73,74,75,76,77,78]. Hayashi et al. [69] studied TE properties of CuCrO_2_ in bulk form with several dopants and reported that the Mg-doped samples showed a higher electrical conductivity leading to a higher power factor (*PF* = 2.36 × 10^−4^ W·m^−1^·K^−2^ at 820 °C) than the undoped bulk. In comparison with CuCrO_2_ and CuCrO_2_:Mg studies carried out on bulk materials [70,71,74,79,80], there is a lack of papers related to proper studies of the TE properties of CuCrO_2_:Mg thin films, as for instance, the determination of *PF*. Most of the time, Seebeck measurements presented in the bibliography are only used to characterize the fundamental physical properties (type of charge carriers, transport mechanism) [16,73,81,82,83,84,85] especially because it is difficult to determine the main charge carrier characteristics by conventional Hall measurements in this type of material. In particular, we have measured the Seebeck coefficient at room temperature for 100 nm thick films in order to determine the type of the main charge carrier in CuCrO_2_:Mg thin films optimized for TCO properties [16]. In the present article, the work is focused on the determination of electrical conductivities and TE properties (Seebeck coefficient and calculated power factor) in temperature ranging from 40 to 220 °C for one set of CuCrO_2_ films annealed at various temperatures, with a fixed thickness of 300 nm.

## 2. Materials and Methods

### 2.1. Preparation of Mg-Doped CuCrO_2_ (Target)

Polycrystalline CuCr_0.97_Mg_0.03_O_2_ powder was prepared by grinding and mixing the starting commercial oxides, Cu_2_O, Cr_2_O_3_, and MgO with appropriate proportions. The stoichiometric oxide mixture was annealed at 900 °C for 10 h in an ambient nitrogen atmosphere and cooled down to room temperature. After it was reground, the mixture was reheated for a further 10 h period. The purity of the phase was checked by X-Ray Diffraction (XRD).

The polycrystalline delafossite powder has been pressed into a sputtering target of 10 cm in diameter then sintered at 1200 °C for 10 h in air. The X-Ray Diffraction (XRD) analysis on a small representative pellet showed only the 3R delafossite phase. (R-3m space group with *a* = 2.9755(2) Å and *c* = 17.091(3) Å as determined by the Rietveld method).

### 2.2. Preparation of Mg-Doped CuCrO_2_ Thin Films

In order to deposit CuCrO_2_:Mg thin films, the target assembly was attached to an Alcatel A450 RF magnetron sputtering chamber (Alcatel, France). Fifteen minutes of pre-sputtering with argon plasma has been applied before starting the film deposition to remove the surface contamination. Pre-cleaned fused silica substrates (25 mm × 25 mm, ≃1 mm thick) placed on a water-cooled sample holder were used during the deposition. In order to avoid the reduction of the target, a low argon pressure [86] was used during the sputtering process. The deposition parameters are summarized in the Table 1. Under these conditions, as-deposited films with normalized thickness of 300 nm were elaborated. The X-ray fluorescence (XRF) measurement (not shown here) carried out with a Bruker S2 apparatus showed that the ratio was close to 1 Cu for 1 Cr in the film (with accuracy range of 5%) which is consistent with the composition of the target (CuCr_0.97_Mg_0.03_O_2_).

The as-deposed films have been systematically annealed for 4 h under primary vacuum at various temperatures ranging from 450 to 650 °C.

### 2.3. Characterization

The structural properties of the films were investigated by a α = 1° grazing incidence X-ray diffraction (GIXRD) at room temperature. GIXRD was performed using a Siemens D5000 diffractometer equipped with a Bruker Sol-X detector (Siemens, Pittsburgh, PA, USA). Copper radiations were used as X-ray source (λCuK_α1_ = 1.5405 Å and λCuK_α2_ = 1.5445 Å). The microstructure of the films was observed using a Nanoscope III Dimension 3000 Atomic Force Microscope (AFM). AFM surface views were analyzed using the Gwyddion software. The band gap energy has been determined by the measure of the total transmittance and the total reflectance in the 300 to 1100 nm wavelength range using a Bentham PVE300 integrated spectrophotometer (Bentham Instruments Ltd., Berkshire, UK).

The electrical resistivity was measured using a four-point probe measurement unit (Signatone, Gilroy, CA, USA).

**Figure 1:**
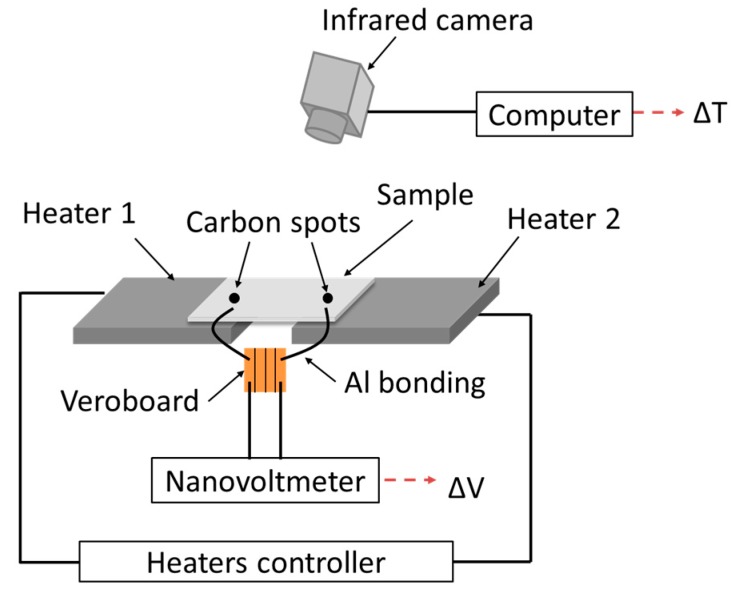
Seebeck coefficient measurement setup.

A home-made measurement setup has been used for the Seebeck coefficient determination as a function of temperature (Figure 1). Two independent heaters fitted to the thin film geometry have been used to apply a thermal gradient along the thin film. Electrical contacts were done with a 25 µm diameter aluminum wire bonder (HYBOND Model 626, Hybond, Escondido, CA, USA). The ohmic type behavior (linearity of current vs. voltage curve) of the electrical contacts has been checked systematically for all samples with a source meter (Keithley 2450, Tektronix, Beaverton, OR, USA) after bonding step. During the experiment, the voltage was measured with a nanovoltmeter (Keithley 2182A, Tektronix, Beaverton, OR, USA). Two carbon spots (emissivity coefficient of 0.97) were deposited on the surface of the thin films by spraying carbon solution through a shadow mask to accurately measure the surface temperature with an infrared camera. The two carbon spots were located at the same isotherm position than the electrical contacts. The mean temperature (*T*_Mean_) was considered as the average between the temperature of the hot side (*T*_Hot_) and that of the cold side (*T*_Cold_).

The Seebeck coefficient *S*(*T*_Mean_) at a given mean temperature can be calculated with

(2) $$S(T_{\mathrm{Mean}})=S_{\mathrm{ref}}-\frac{\Delta V}{\Delta T}$$

where *S*_ref_, Δ*V*, and Δ*T* are respectively Seebeck coefficient of the reference (Aluminum probe: *S* = 3.5 µV·K^−1^), electric potential and temperature difference (*T*_Hot_ − *T*_Cold_) measured on the film. The accuracy of the experimental setup was checked by using a bar of Ca_3_Co_4_O_9_ already measured elsewhere with a ZEM3 commercial apparatus. The results were similar with a standard deviation of 7%.

## 3. Results and Discussion

### 3.1. Structural and Microstructural Characterizations

**Figure 2:**
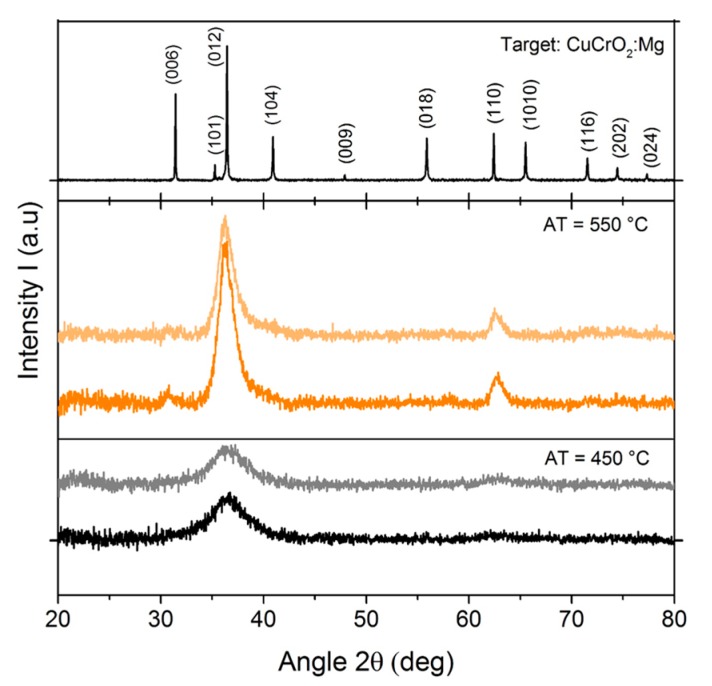
XRD pattern of the target, GIXRD patterns registered at room temperature (α = 1°) after annealing treatment (at 550 and 450 °C) (dark color) and GIXRD patterns of the same annealed samples registered after a thermal treatment at 240 °C under air atmosphere (light color).

The GIXRD patterns of CuCrO_2_:Mg thin films annealed for 4 h at various temperature in the 450 to 650 °C temperature range under primary vacuum have been measured for all samples. The as-deposited sample and the sample annealed at 450 °C were amorphous or nanocrystallized. The samples annealed above 500 °C corresponded to the target pattern of CuCrO_2_ verifying the rhombohedral R-3m space group [87] (Figure 2). The full width at half maximum (FWHM) for the peak (012) decreased strongly with increasing annealing temperature (*AT*), up to 550 °C. For *AT* above 550 °C, the FWHM value was stable. As the instrumental contribution and the micro-strain are constant or negligible, it means that grain growth mainly occurred between 450 and 550 °C. The GIXRD pattern of the sample annealed at 450 and 550 °C were shown as an example in the Figure 2. All the characteristic Bragg peaks of the 3R delafossite phase are reported (PC-PDF file #39-0247). It confirms that the experimental conditions used to elaborate the samples provided a pure CuCrO_2_:Mg phase. The lattice parameters determined by profile matching with constant scale factor were *a* = 2.96(7) Å and *c* = 17.14(5) Å.

In order to check the stability of the delafossite phase during the various measurements in temperature, a GIXRD analysis was done after a thermal treatment at 240 °C under air atmosphere for all samples. The result (Figure 2) shows similar patterns before (dark color) and after the thermal treatment for thin film initially annealed at 450 and 550 °C (light color). Thus, there were no additional phases formed during the physical characterization in temperature from room temperature to 220 °C in air atmosphere.

**Figure 3:**
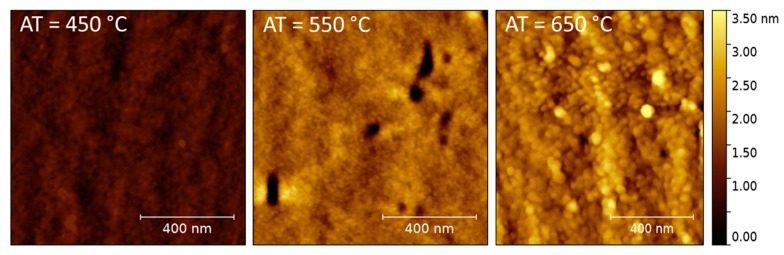
AFM micrographs of CuCrO_2_:Mg thin films annealed at 450, 550, and 650 °C for 4 h under primary vacuum.

The AFM analysis of the CuCrO_2_:Mg annealed at 450, 550, and 650 °C reveals the microstructure of the thin films (Figure 3). They exhibit nanocrystallized surfaces. The results show a smooth surface for the 450 °C and 550 °C annealed films and for 650 °C annealed film, the grains are well-defined. Hence, the AFM results are in perfect agreement with the GIXRD analysis.

### 3.2. Transport Properties

**Figure 4:**
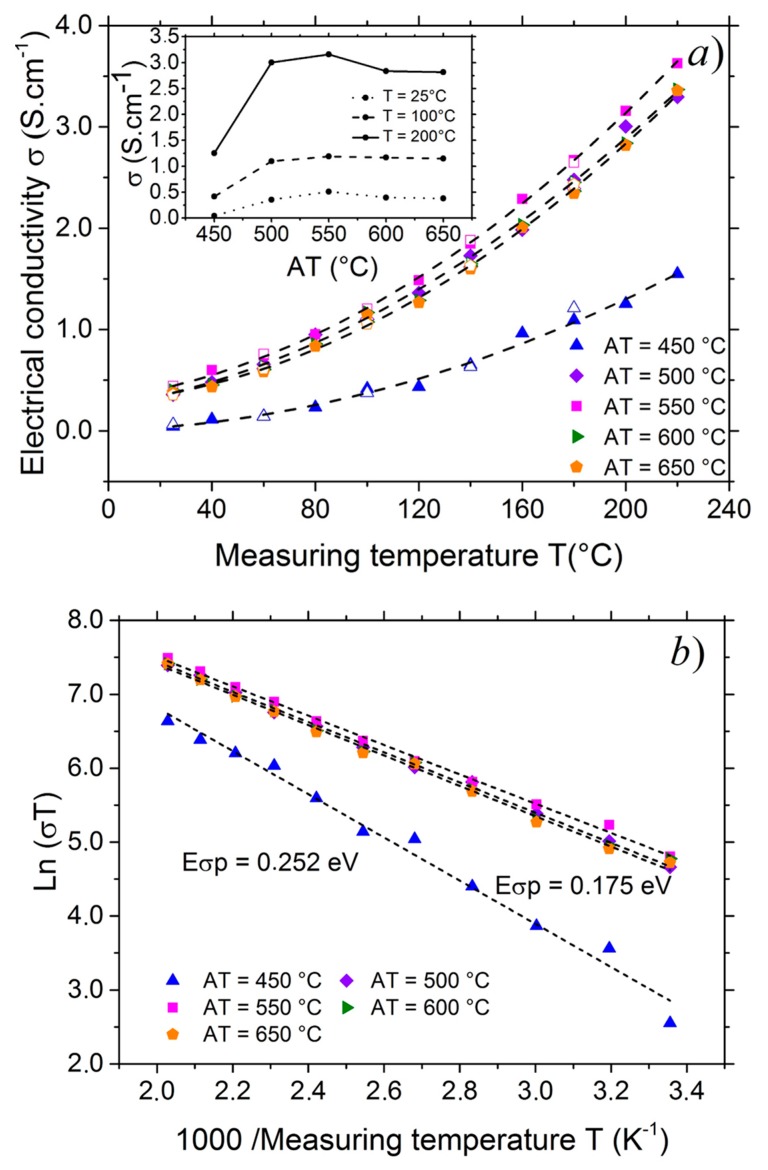
(**a**) Electrical conductivity σ of the films as a function of the measuring temperature *T* while heating (filled symbol) and cooling (empty symbol). Inset: Electrical conductivity *σ* of the films as a function of annealing temperatures (*AT*) at three different measuring temperatures; (**b**) Arrhenius plot of the electrical conductivity *σ*.

The inset of the Figure 4a shows the electrical conductivity σ, at three measuring temperatures, of the 300 nm thick films as a function of the annealing temperature (*AT*). The as-deposited sample had the lowest electrical conductivity (at room temperature *σ*_as-deposited_ = 6.10^−3^ S·cm^−1^). This electrical conductivity measured at room temperature increased with the annealing temperature from 450 to 550 °C and reached 0.51 S·cm^−1^ then decreased slightly for higher annealing temperatures. Similar decrease of the electrical conductivity has already been observed by various authors and attributed to the decrease of copper vacancies and oxygen interstitials at higher temperatures [75,88] or microstructural changes [16]. In the literature, the electrical conductivity of CuCrO_2_:Mg films ranged from 0.033 to 1.6 S·cm^−1^ [16,78,83,84,89,90]. For 305 nm thick CuCrO_2_:Mg films, Rastogi et al. [84] found 0.1–0.2 S·cm^−1^ whereas, higher electrical conductivity of 0.6–1 S·cm^−1^ was obtained for 155 nm thick films. Their results are consistent with the present study.

The Figure 4a,b shows the variation of the electrical conductivity of the CuCrO_2_:Mg films with the measuring temperature. The electrical measurements showed that the variation of the electrical conductivity was fully reversible and increased with temperature as with semiconducting oxides.

For a semiconductor, the relation of the electrical conductivity with the temperature can be written in two ways considering that thermal energy is enough to activate the carriers (corresponding to the classical conduction mode) or not (corresponding to the hopping mechanism). In the second case, the thermal energy activates the small polarons and σ can be expressed as Equation (3). CuCrO_2_ is known as a polaronic material where the transport mechanism is limited by small polaron hopping (SPH) [72,91,92,93,94].

In the case of small polarons, the expression of electrical conductivity is given by Mott and Davis [95]

(3) $$\sigma =\frac{A_{\sigma p}}{T}e^{-\frac{E_{\sigma p}}{k_{B}T}}$$

where *A_σp_* is a constant, *E_σp_* is the activation energy of polaronic conduction.

In order to calculate the activation energy *E_σp_* using SPH model, we plotted ln(*σT*) versus 1000/*T* in the Figure 4b. The results showed a linear variation and the slope of the lines gave *E_σp_*/*k_B_*. It is found that the polaronic activation energy decreased when annealing was carried out above 500 °C and reached a quasi-constant value close to 175 meV.

### 3.3. Seebeck Coefficient Measurement

**Figure 5:**
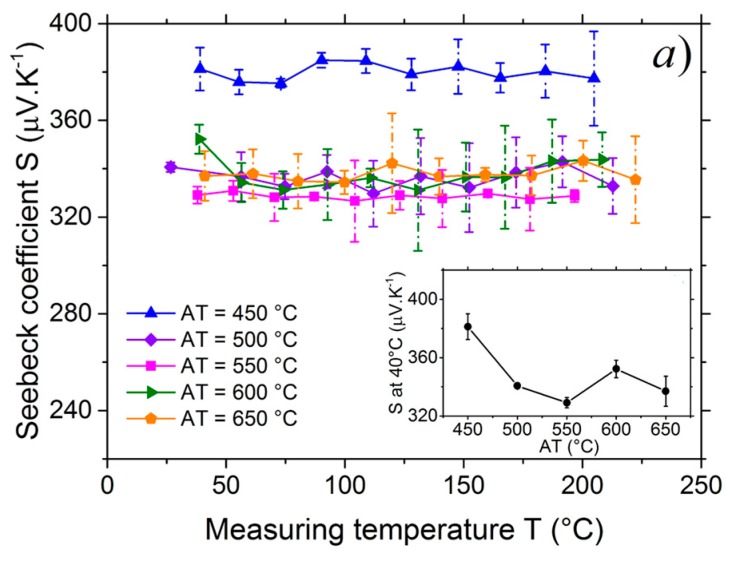

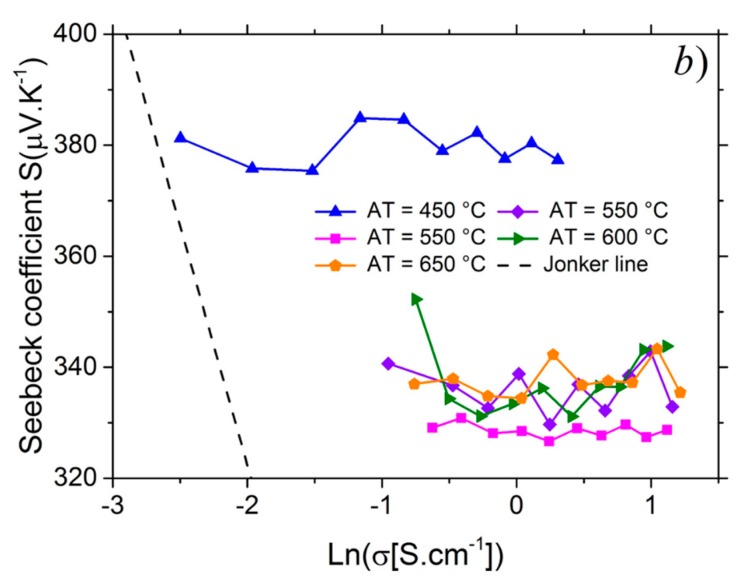
(**a**) Seebeck coefficient, *S*, of the films annealed at different temperatures as a function of the measuring temperature, *T*. Inset: Seebeck coefficient, *S*, of the films at 40 °C as a function of the annealing temperature, *AT*; (**b**) Jonker plot: the relationship of the Seebeck coefficient, *S*, to the electrical conductivity σ for different annealing temperatures, *AT*.

The Figure 5a shows the Seebeck coefficient of the films as a function of the measuring temperature. The positive values of Seebeck coefficient confirmed that the CuCrO_2_:Mg films were p-type semiconductors for all annealing temperatures. The lowest Seebeck coefficient was obtained for the highest electrical conductivity due to the carrier density variation. The measurement temperature, from 40 to 220 °C, did not influence the Seebeck coefficient. In the literature, the Seebeck coefficient of CuCrO_2_:Mg bulk is ranging between 200–500 µV·K^−1^ and does not vary a lot with temperature in the 77 to 827 °C range [69,70,79] which is coherent with the present Seebeck measurements. Tripathi et al. [82] reported comparable value (≃+300 µV·K^−1^ at room temperature) for undoped CuCrO_2_ films. On the other hand, Chikoidze et al. [83] published three times lower value of Seebeck coefficient (+130 µV·K^−1^ at 23 °C) for 4% Mg doped CuCrO_2_ thin film. Overall, trends are similar and the difference in values could be explained by the difference in the measurement setup and the material type.

The invariance of Seebeck coefficient in temperature indicates that the hole density is constant between 40 and 220 °C. Therefore, the increase in electrical conductivity is the consequence of the increase in small polarons mobilities due to the hopping mechanism. In the classical conduction mode within the saturation regime, the electrical conductivity would have decreased because of the carrier mobility diminution ($\mu \propto T^{\frac{-3}{2}}$).

Jonker relation (Equation (4)) shows that Jonker plot *S* versus ln(*σ*) would have a slope *k_B_*/*q* = ±86.17 µV·K^−1^ indicating the thermally activated non-degenerate semiconductor behavior (free carrier).

(4) $$S=\pm \frac{k_{B}}{q}(\ln\sigma -\ln\sigma_{0})$$

The plotted curves of the films in the inset of the Figure 5b were far away from the Jonker line. Consequently the Jonker plot *S* versus ln(*σ*) [96] showed a degenerate semiconductor behavior for the whole samples. Chikoidze et al. [83] noticed the quasi invariance of the Seebeck coefficient with the measurement temperature, which is consistent with the present results. Farrell et al. [72] also reported degenerate behavior for Cu deficient CuCrO_2_ film.

### 3.4. Power Factor (PF)

**Figure 6:**
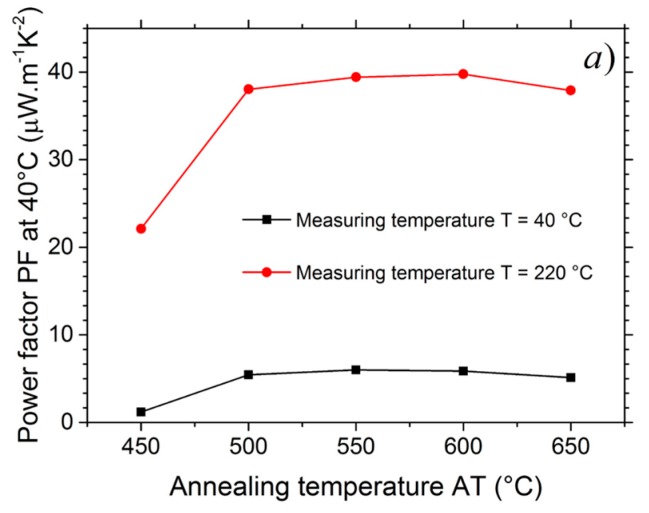

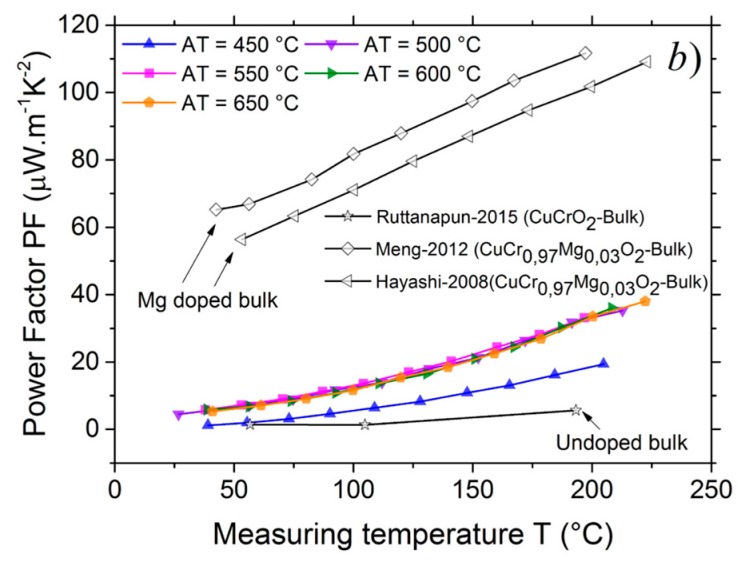
(**a**) Power factor *PF* at 40 and 220 °C as a function of the annealing temperature *AT*; (**b**) Power factor *PF* of annealed thin films as a function of the measuring temperature *T* and comparison with the data from the literature.

The *PF* values were calculated from the electrical conductivity and the Seebeck coefficient (*PF* = *σS*^2^). The Figure 6a shows the variation of the *PF* at 40 °C as a function of the annealing temperature and the highest *PF* is obtained for samples annealed between 550 and 600 °C. The Figure 6b shows the power factor (*PF*) for the CuCrO_2_:Mg films annealed at different temperatures, as a function of the measuring temperature and gives a comparison with the data from the literature. As the Seebeck coefficient was quasi constant with increasing temperature, the *PF* followed the electrical conductivity variation. It increased with increasing measurement temperature. Around 220 °C, it reached 38 µW·m^−1^·K^−2^. CuCrO_2_:Mg films had a higher *PF* than the CuCrO_2_ bulk studied by Ruttanapun et al. [80]. However, their *PF* were lower than the experimentally achieved *PF* of CuCrO_2_:Mg bulk materials [69]. For example, Q. Meng et al. found 65 µW·m^−1^·K^−2^ at 40 °C for CuCrO_2_:Mg bulk material [79] thanks to the high electrical conductivity. In comparison with the *PF* obtained with bulk material, a slight improvement of the power factor of CuCrO_2_:Mg in the form of thin film could still be achieved by optimizing the film nanostructuration. Moreover, the thickness of the films deposited by RF-magnetron sputtering were 300 nm and showed columnar nanometric grains [16] which implies a lower thermal conductivity due to phonon boundary scattering without reducing the electrical conductivity.

The main advantage of the thin film compared to the bulk is noticeable with the figure of merit *ZT* where the thermal conductivity is taken into account. Indeed, the lattice thermal conductivity in thin films is generally lower than the bulk materials [2,7,8]. However, in this work, the values for *ZT* have not been calculated because the thermal conductivity of CuCrO_2_:Mg in the form of thin films could not be measured.

### 3.5. Optoelectrical Properties and Fermi Energy Level

**Figure 7:**
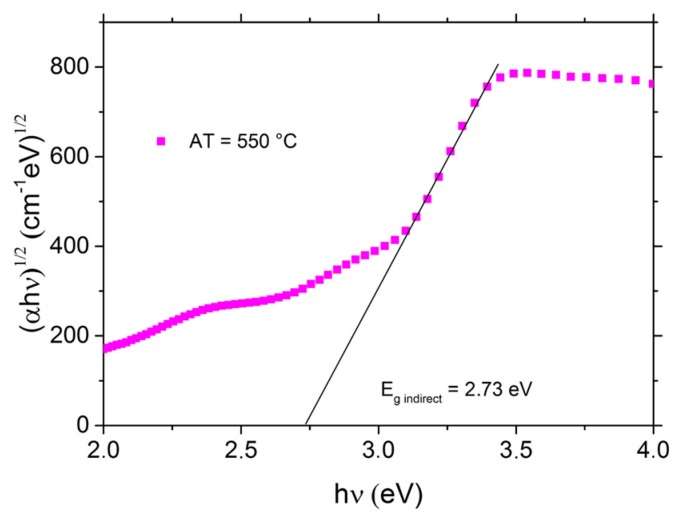
(αh*ν*)^1/2^ vs. h*ν* plot for the fundamental indirect optical band gap energy analysis.

The fundamental indirect band gap energy was determined by the measurements of the total transmittance *TT* (specular and diffuse) and the total reflectance *TR* (not shown here). The Tauc’s plot [97] gives the optical fundamental band gap from the linear extrapolation of the curve slope to intercept the energy axis (Figure 7). In detail, the absorption coefficient *α*, was estimated from the relation [98]

(5) $$\alpha =\frac{1}{d}\ln\left(\frac{{\left(1-TR\right)}^{2}}{TT}\right)$$

where *d* is the film thickness. The fundamental indirect band gap energy was determined using the absorption coefficient α via the relation

(6) $${\left(\alpha h\upsilon \right)}^{m}=A\left(h\upsilon -E_{g}\right)$$

where h*ν* is the photon energy, *E*_g_ is the optical band gap, *A* is a constant called the band tailing parameter and *m* = 1/2 for the fundamental indirect transition. Using the Tauc’s method, 2.73 eV was obtained for the fundamental indirect transition (Figure 7). The obtained energy value is consistent with the literature [84] and shows a wide band gap which avoids the simultaneous presence of the electron and hole in their specific bands and drives to a high Seebeck coefficient even at high temperatures. A similar indirect band gap value was obtained by Chikoidze et al. [83] and Kaya et al. [99].

The Fermi energy was calculated using the following Mott formula [100] for a degenerate electron gas approximation

(7) $$S\approx -\frac{\pi^{2}k_{B}^{2}T}{3qE_{F}}$$

In this case, the Fermi energy is taken from the valence band edge. At 40 °C, the Fermi energy value was 0.022 ± 0.002 eV which is lower than the thermal energy (3*k_B_T* = 0.081 eV at 314 K) on the Fermi distribution width. It leads to the conclusion that the samples did not display thermally activated characteristics, which is consistent for a degenerate semiconductor. The Fermi level can trace the work function of the delafossite and give precious information about which metal can be used for ohmic contact (to avoid the Schottky barriers) with the delafossite. This information is needed for the thermoelectric, optoelectronic, and photocatalytic applications.

### 3.6. Carrier Concentration and Hole Effective Mass

According to the Hubbard model [101] and Heike’s formula [102] adapted for degenerate semiconductor with a carrier hopping mechanism, the Seebeck coefficient can be written as Equation (8)

(8) $$S=+\frac{k_{B}}{q}\left[\left(\frac{g_{1}}{g_{2}}\right)\frac{\left[Cu^{+}\right]}{\left[Cu^{2+}\right]}\right]$$

where *g* is the spin and orbital degeneracy and [Cu^+^]/[Cu^2+^] is the ratio of the ion concentrations.

**Figure 8:**
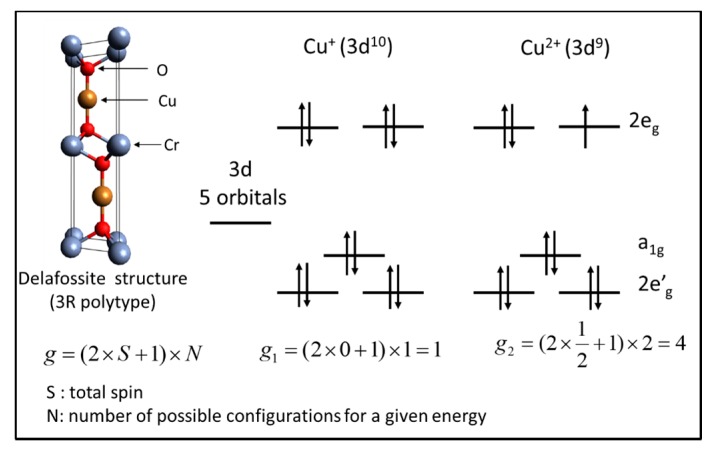
Spin and orbital degeneracies of Cu^+^ and Cu^2+^ in the delafossite structure.

At high temperature, due to the mixed valences of the copper (Cu^+^/Cu^2+^) in the CuCrO_2_:Mg, the Seebeck coefficient which is the measure of the entropy of the carriers is given by the spin and orbital degeneracy [73]. The Figure 8 shows the spin and orbital degeneracies of Cu^+^ (*g*_1_) and Cu^2+^ (*g*_2_) due to Mg^2+^ doping compensation according to (Cu^+^_1−*x*_Cu^2+^*_x_*)(Cr^3+^_1−*x*_Mg^2+^*_x_*)O_2_ in the linear bonding O–Cu–O (where the coordination number of Cu is 2). Thereby, from the measured Seebeck coefficient, the concentration of Cu^+^ and Cu^2+^ ions have been estimated and they varied barely with the annealing temperature (*AT*). The obtained values were [Cu^+^] = 0.995 and [Cu^2+^] = 0.005 for *AT* above 500 °C.

Using the unit cell volumes determined by the GIXRD measurements, the density of total copper site *d*_Cu_ in the delafossite structure is estimated at (2.30 ± 0.02) × 10^22^ cm^−3^. The hole density which is related to the Cu^2+^ concentration is equal to *d*_Cu_ × [Cu^2+^] = (1.17 ± 0.05) × 10^20^ cm^−3^.

The hole effective mass, m*, was calculated using the equation

(9) $$m^{*}=\frac{\hbar^{2}}{2E_{F}}{\left(3\pi^{2}h_{s}\right)}^{2/3}$$

where *ћ* is the reduced Planck constant and *h*_s_ is the hole density.

The hole effective mass estimated for CuCrO_2_:Mg was 3.80 ± 0.2 *m*_0_ which is close to the effective mass predicted theoretically for CuCrO_2_ by Scanlon et al. [103] (without spin-orbit coupling m*_th_ = 2.96 *m*_0_ and with spin-orbit coupling m*_th_ = 4.53 *m*_0_).

The effective hole mass is rarely published for this type of material. However, it is requisite for the optoelectronic application in particular for the determination of the hole mobility.

## 4. Conclusions

CuCrO_2_:Mg thin films have been elaborated by RF magnetron sputtering and annealed at different temperatures between 450 and 650 °C under primary vacuum. The as-deposited film and the film annealed at 450 °C were nanocrystalized, whereas the films annealed above 500 °C had a delafossite structure. The AFM has shown a nanometric grains sizes and smooth surface at the optimal annealing temperature. The higher electrical conductivity determined at 40 °C was 0.60 S·cm^−1^ for the film annealed at 550 °C which had a Seebeck coefficient of +329 µV·K^−1^. We analyzed the small polaron hopping conductivity mechanism and found a degenerate semiconductor behavior of the CuCrO_2_:Mg thin films. Thanks to its constant Seebeck coefficient between 40 and 220 °C and high fundamental indirect band gap energy, the power factor increased like the electrical conductivity when the temperature was increased, and reached 38 µW·m^−1^·K^−2^ at 220 °C. CuCrO_2_:Mg thin films have been studied for their TE properties and showed encouraging results. Their high and constant Seebeck coefficient in changing temperatures and their stability in air atmosphere could be a great advantage for an application of this material in high accuracy temperature measurement devices, miniaturized devices in thin film configuration, and also transparent TE devices due to its TCO properties.

## Figures and Tables

**Table 1:** Process parameters for the deposition of delafossite Mg-doped CuCrO_2_ by RF-sputtering.

Target material	3 at % Mg-doped CuCrO_2_
Substrate	Fused quartz
Power (W·cm^−2^)	0.9
Magnetron	Yes
Argon pressure P (Pa)	0.5
Target to substrate distance *d* (cm)	5
