# Correction: Sinnarasa, I.; et al. Thermoelectric and Transport Properties of Delafossite CuCrO_2_:Mg Thin Films Prepared by RF Magnetron Sputtering. *Nanomaterials* 2017, *7*, 157

**DOI:** 10.3390/nano7110357

**Published:** 2017-10-30

**Authors:** Inthuga Sinnarasa, Yohann Thimont, Lionel Presmanes, Antoine Barnabé, Philippe Tailhades

**Affiliations:** CIRIMAT, Université de Toulouse, CNRS, INPT, UPS, 118 route de Narbonne, F-31062 Toulouse CEDEX 9, France; sinnarasa@chimie.ups-tlse.fr (I.S.); presmanes@chimie.ups-tlse.fr (L.P.); barnabe@chimie.ups-tlse.fr (A.B.); tailhades@chimie.ups-tlse.fr (P.T.)

The authors wish to make the following correction to this paper [1]. In Equation (8), logarithm (ln) term is missing.

Here, we supply the corrected Equation (8).

(8) $$S=+\frac{k_{B}}{q}\ln\left[\left(\frac{g_{1}}{g_{2}}\right)\frac{\left[Cu^{+}\right]}{\left[Cu^{2+}\right]}\right]$$
